# Dr. Henry Oliver Ely

**Published:** 1917-06

**Authors:** 


**Dr. Henry Oliver Ely,** P. & S. (now Columbia) 1867, died at his home in Binghamton, April 18, aged 71.

